# Supplementary material for: Changes in Alcohol Consumption and Risk of Heart Failure: A Nationwide Population-Based Study in Korea
Source: Int J Environ Res Public Health. 2022 Dec 5;19(23):16265. doi: 10.3390/ijerph192316265 (PMC9736316; doi:10.3390/ijerph192316265)
Supplement: Supplementary file 1 [file ijerph-19-16265-s001.zip › ijerph-1940159-supplementary Table.pdf]

Supplementary Table S1. Summary of previous longitudinal studies identifying the association between alcohol intake and heart failure risk

Study population	Non-drinkers (Abstainers / Former drinkers)	Light to moderate drinkers		Heavy drinkers
	HR (95% CI)	HR (95% CI)		HR (95% CI)
1. Abramson et al., 2001 [1]				
[Men + Women] Aged >65, US<br>Total N = 2,235,<br>HF cases = 281<br>Mean f/u 14 years	**(Ref: non-drinker)**	<1.5 drinks/day<br>: 0.79 (0.60–1.02)	1.5–4 drinks/day<br>: 0.53 (0.32–0.88)	
2. Aguilar et al., 2004 [2]				
[Men + Women] Aged 21-80, US, Canada<br>Total N = 2228,<br>HF cases = 456<br>Mean f/u of 3.5 years	**(Ref: non-drinker)**	0–10 drinks/week<br>: 0.93 (0.75–1.17)	>10 drinks/week<br>: 1.25 (0.91–1.72)	
3. Bryson et al., 2006 [3]				
[Men + Women] Aged ≥65, US<br>Total N = 4,368<br>HF cases = 674<br>Mean f/u of 7-10 years	**(Ref: abstainers)**<br>Former drinkers<br>1.47 (1.13–1.92)	<1 drink/week<br>0.88 (0.70–1.10)<br><br>1–6 drinks/week<br>0.82 (0.64–1.06)	7–13 drinks/week<br>0.72 (0.48–1.08)	≥14 drinks/week<br>: 0.97 (0.70–1.33)
4. Djoussé et al., 2007 [4]				
[Men] aged 40-85, USA<br>Total N = 21, 601<br>HF cases = 904<br>Mean f/u of 18.4 years	-	**(Ref: <1 drink/week)**<br><br>1–4 drinks/week<br>: 0.90 (0.76–1.07)	5–7 drinks/week<br>: 0.84 (0.71–0.99)	>7 drinks/week<br>: 0.62 (0.41–0.96)
5. Dorans et al, 2015 [5]				
[Men] aged 45-79, Sweden<br>Total N = 33,760<br>HF cases = 2,916<br>Mean f/u of 13 years	**(Ref: non-drinker)**	<0.5 drinks/week<br>: 1.04 (0.85, 1.28)<br><br>0.5 – <1 drinks/week<br>: 0.86 (0.70, 1.07)<br><br>1-<7 drinks/ week<br>: 0.89 (0.76, 1.04)	7-<14 drinks/ week<br>: 0.81 (0.69, 0.96)<br><br>14-<21 drinks/ week<br>: 0.81 (0.65, 1.02)	>21 drinks/ week<br>: 1.12 (0.85, 1.47)
6. Gemes et al, 2016 [6]				
[Men + Women] Norway<br>Total N = 60, 665<br>HF cases = 1,588<br>Mean f/u of 11.2 years	**(Ref: abstainers and rare drinkers, i.e. ≤0.5 drink/week)**	>0.5 and ≤3 drinks/week<br>: 0.83 (0.72–0.96)<br><br>>3 and ≤5 drinks/week	>5 and ≤7 drinks/week<br>: 0.81 (0.53–1.22)	>7 drinks/weeks<br>: 0.84 (0.51–1.24)

		: 0.67 (0.50–0.92)		
7. Goncalves et al, 2015 [7]				
[Men] US<br>Total N= 6,583<br>HF cases = 1,237<br>Total f/u of 24 years	**(Ref: abstainers)**<br><br>Former drinkers<br>: 1.19 (1.02–1.39)	<7 drinks/week<br>: 0.80 (0.68–0.94)	7-14 drinks/week<br>: 0.92 (0.75–1.13)<br><br>14-21 drinks/week<br>: 0.86 (0.65–1.13)	>21 drinks/week<br>: 0.98 (0.75–1.28)
[Women] US<br>Total N = 8,046<br>HF cases = 1,271<br>Total f/u of 24 years	**(Ref: abstainers)**<br><br>Former drinkers<br>: 1.17 (1.01–1.36)	<7 drinks/week<br>: 0.84 (0.71–1.00)	7-14 drinks/week<br>: 0.93 (0.68–1.27)<br><br>14-21 drinks/week<br>: 1.24 (0.72–2.12)	>21 drinks/week<br>: 0.78 (0.29–2.09)
8. Janszky et al., 2008 [8]				
[Men + Women] aged 45-70, Sweden<br>Total N = 1,346<br>HF cases = NA<br>Mean f/u of 8 years	**(Ref: abstainers)**<br><br>Recent quitters<br>2.51 (1.11–5.65)	>0 to <5 g/day<br>0.74 (0.53–1.04)	5–20 g/day<br>0.84 (0.59–1.20)	>20 g/day<br>0.91 (0.61–1.34)
9. Klatsky et al., 2005 [9]				
[CAD-HF] [Men + Women] USA<br>Total N = 126,236<br>HF cases = 1559<br>Mean f/u of 14.4 years	**(Ref: abstainers)**<br><br>Former drinkers<br>1.2 (1.0–1.5)	<1 drink/month<br>: 0.9 (0.8–1.0)<br><br><1 drink/day<br>: 0.7 (0.6–0.8)	1–2 drinks/day<br>: 0.6 (0.5–0.7)<br><br>3–5 drinks/day<br>: 0.6 (0.5–0.8)	≥6 drinks/day<br>: 0.5 (0.3–0.8)
[Non-CAD-HF] [Men + Women] USA<br>Total N = 126, 236<br>HF cases = 1035<br>Mean f/u of 14.4 years	**(Ref: abstainers)**<br><br>Former drinkers<br>1.0 (0.7–1.3)	<1 drink/month<br>: 0.9 (0.7–1.1)<br><br><1 drink/day<br>: 0.8 (0.7–1.0)	1–2 drinks/day<br>: 1.0 (0.8–1.3)<br><br>3–5 drinks/day<br>: 1.2 (0.9–1.6)	≥6 drinks/day<br>: 1.7 (1.1–2.6)
10. Larsson et al., 2015 [10]				
A meta-analysis of 8 prospective studies<br>Total N = 202, 378<br>HF cases = 6,211	**(Ref: non-drinker)**	Light to moderate<br>: 0.85 (0.78-0.93)		Heavy<br>: 0.90 (0.72-1.13)
11.Larsson et al, 2017 [11]				
[Men] Sweden<br>Total N = 40,590<br>HF cases = 1,905<br>Mean f/u of 12 years	Never<br>: 1.27 (1.02–1.58)<br><br>Former<br>: 1.36 (1.12–1.66)	**(Ref: Current, <1 drinks/week)**<br><br>1–6 (3.7) drinks/week<br>: 1.08 (0.92–1.26)	7–14 (9.8) drinks/week<br>: 1.14 (0.95–1.36)<br><br>15–21 (16.6) drinks/week<br>: 0.94 (0.74–1.20)<br><br>22–28 (23.8)	>28 (37.4) drinks/week<br>1.45 (1.09–1.93)

			drinks/week<br>: 1.20 (0.87–1.65)	
[Women] Sweden<br>Total N = 34, 022<br>HF cases = 1328<br>Mean f/u of 12 years	Never<br>: 1.05 (0.90–1.22)<br><br>Former<br>: 1.11 (0.84–1.48)	**(Ref: Current, <1 drinks/week)**<br><br>1–6 (3.0) drinks/week<br>: 0.93 (0.81–1.07)	7–14 (9.2) drinks/week<br>: 0.96 (0.74–1.24)<br><br>15–21 (16.4) drinks/week<br>: 0.68 (0.32–1.44)	>21 (26.1) drinks/week<br>: 0.74 (0.33–1.67)
12. Walsh et al., 2002 [12]				
[Men] Aged 28-62, US<br>Total N = 2,796<br>HF cases = 99<br>Mean f/u 10.2 years	**(Ref: non-drinker)**<br><br>Former drinkers<br>0.64 (0.34–1.22)	1–7 drinks/week<br>: 0.46 (0.27–0.81)	8–14 drinks/week<br>: 0.47 (0.24–0.94)	≥15 drinks/week<br>: 0.63 (0.34–1.19)
[Men] [Non-CAD HF] Aged 28-62, US<br>Total N = 2,796<br>HF cases = 71<br>Mean f/u 10.2 years	**(Ref: non-drinker)**<br><br>Former drinkers<br>0.55 (0.25–1.22)	1–7 drinks/week<br>: 0.43 (0.22–0.83)	8–14 drinks/week<br>: 0.56 (0.26–1.22)	≥15 drinks/week<br>: 0.75 (0.36–1.53)
[Women] Aged 28-62, US<br>Total N = 3493,<br>HF cases = 120<br>Mean f/u 10.2 years	**(Ref: non-drinker)**<br><br>Former drinkers<br>1.15 (0.72–1.86)	1–2 drinks/week<br>: 0.82 (0.48–1.39)	3–7 drinks/week<br>: 0.60 (0.31–1.18)	≥8 drinks/week<br>: 1.04 (0.56–1.92)
[Women] [Non-CAD HF] Aged 28-62, US<br>Total N = 3493,<br>HF cases = 85<br>Mean f/u 10.2 years	**(Ref: non-drinker)**<br><br>Former drinkers<br>0.95 (0.51–1.75)	1–2 drinks/week<br>: 1.13 (0.63–2.01)	3–7 drinks/week<br>: 0.60 (0.27–1.33)	≥8 drinks/week<br>: 1.02 (0.49–2.10)
13. Wang et al., 2011 [13]				
[Men] aged 27-74, Finland<br>Total N = 18, 346<br>HF cases = 638<br>Mean f/u of 14.1 years	**(Ref: non-drinker)**	0.1–35 g/week<br>: 0.91 (0.70–1.18)	35.1–70 g/week<br>: 1.00 (0.79–1.27)	>70 g/week<br>: 1.17 (0.96–1.42)
[Women] aged 27-74, Finland<br>Total N = 19,729<br>HF cases = 445<br>Mean f/u of14.1 years	**(Ref: non-drinker)**	0.1–35 g/week<br>: 0.90 (0.67–1.21)	35.1–70 g/week<br>: 1.01 (0.68–1.50)	>70 g/week<br>: 0.92 (0.53–1.57)
14. Wannamethee et al, 2015 [14]				
[Men] aged 60-79, UK<br>Total N = 3,530<br>HF cases = 198<br>Mean f/u of 11 years	Non-drinker<br>: 0.97 (0.59 to 1.63)	<1 drinks/week<br>: 1.39 (0.86 to 2.25)<br><br>**(Ref: 1-6 drinks/week)**	7-14 drinks/week<br>: 0.94 (0.64 to 1.43)<br><br>15-34 drinks/week<br>: 1.16 (0.78 to 1.71)	>35 drinks/week<br>: 1.91 (1.02 to 3.56)

HR, hazard ratio; CI, confidence interval; N, number; HF, heart failure; CAD, coronary artery disease

Supplementary Table S2. Hazard ratios (HR) and 95% confidence intervals (CI) for the association between change in alcohol consumption amount and the risk of heart failure

		Development of heart failure				Abstainers as a reference		Sustainers at the same level of drinking as a reference	
2009 (First)	2011 (Second)	N	Case No.	Follow-up (Person-years)	IR (1000 person-years)	Crude HR (95% CI)	aHR (95% CI)†	Crude HR (95% CI)	aHR (95% CI)†
Non	Non	1,801,711	57,553	11,381,089.97	5.06	1(Ref.)	1(Ref.)	1(Ref.)	1(Ref.)
	Light	239,304	5528	1,510,818.61	3.66	1.02 (0.99,1.05)	0.92 (0.90,0.95)	1.02 (0.99,1.05)	0.91 (0.89,0.94)
	Moderate	34,149	1035	214,240.04	4.83	1.32 (1.24,1.40)	1.00 (0.94,1.06)	1.33 (1.25,1.41)	0.97 (0.92,1.04)
	Heavy	19,986	717	124688.3	5.75	1.47 (1.37,1.58)	1.05 (0.98,1.13)	1.48 (1.37,1.59)	1.02 (0.95,1.10)
Light	Non	248,072	6,715	1,564,393.88	4.29	1.06 (1.04,1.09)	0.98(0.96,1.01)	1.11 (1.08,1.15)	1.19 (1.15,1.23)
	Light	616,935	11,833	3,896,343.58	3.04	0.96 (0.94,0.98)	0.80 (0.79,0.82)	1(Ref.)	1(Ref.)
	Moderate	128,234	2,833	807,982.89	3.51	1.11 (1.07,1.15)	0.83 (0.80,0.86)	1.16 (1.11,1.21)	1.05 (1.00,1.09)
	Heavy	38,438	1,124	241,140.7	4.66	1.30 (1.22,1.38)	0.93 (0.88,0.99)	1.36 (1.28,1.44)	1.19 (1.12,1.26)
Moderate	Non	35,445	1,338	221,368.56	6.04	1.44 (1.36,1.52)	1.16 (1.10,1.23)	1.25 (1.18,1.34)	1.37 (1.28,1.46)
	Light	148,992	3,504	937,100.04	3.74	1.15 (1.11,1.19)	0.86 (0.83,0.90)	0.99 (0.95,1.04)	1.03 (0.98,1.08)
	Moderate	166,252	3,569	1,046,152.35	3.41	1.17 (1.13,1.21)	0.84 (0.81,0.87)	1(Ref.)	1(Ref.)
	Heavy	73,983	1,965	464,352.09	4.23	1.35 (1.29,1.41)	0.94 (0.90,0.99)	1.16 (1.10,1.23)	1.13 (1.07,1.19)
Heavy	Non	21,970	1,050	135,581.1	7.74	1.69 (1.59,1.79)	1.31(1.23,1.39)	1.20 (1.12,1.29)	1.31 (1.22,1.41)
	Light	48,770	1,565	305,094.17	5.13	1.42(1.35,1.49)	1.03 (0.98,1.08)	1.00 (0.94,1.06)	1.05 (0.99,1.11)
	Moderate	81,994	2,101	513,777.93	4.09	1.27 (1.22,1.33)	0.89 (0.85,0.93)	0.89 (0.84,0.93)	0.90 (0.86,0.95)
	Heavy	138615	4,181	867,275.27	4.82	1.43 (1.39,1.48)	0.98 (0.95,1.01)	1(Ref.)	1(Ref.)

aHR, adjusted hazard ratio; CI, confidence interval; IR, incidence rate

† Adjusted for age, sex, body mass index, smoking status, physical activity, area of residence, income, hypertension, diabetes mellitus, dyslipidemia, systolic blood pressure, fasting glucose level, total cholesterol level, and serum creatinine level

Supplementary Table S3. Adjusted hazard ratios and 95% confidence intervals for the association between change in alcohol consumption amount and the risk of congestive heart failure according to age, sex and smoking status

		**Abstainers as a reference**				**Sustainers at the same level of drinking as reference**			
	**2011 (Second)**	Non	Light	Moderate	Heavy	Non	Light	Moderate	Heavy
	**2009 (First)**	aHR (95% CI)	aHR (95% CI)	aHR (95% CI)	aHR (95% CI)	aHR (95% CI)	aHR (95% CI)	aHR (95% CI)	aHR (95% CI)
Age<65	Non	1(Ref.)	0.93 (0.90,0.97)	0.97 (0.89,1.05)	1.03 (0.93,1.13)	1(Ref.)	0.91 (0.88,0.95)	0.92 (0.85,1.00)	0.98 (0.88,1.08)
	Light	0.98 (0.95,1.02)	0.81 (0.78,0.83)	0.82 (0.78,0.86)	0.92 (0.85,0.99)	1.18 (1.13,1.23)	1(Ref.)	1.03 (0.98,1.09)	1.16 (1.08,1.26)
	Moderate	1.17 (1.09,1.26)	0.86 (0.83,0.90)	0.82 (0.79,0.86)	0.91 (0.86,0.96)	1.37 (1.26,1.49)	1.03 (0.98,1.09)	1(Ref.)	1.11 (1.04,1.19)
	Heavy	1.24 (1.14,1.36)	1.01 (0.94,1.07)	0.86 (0.81,0.91)	0.97 (0.93,1.01)	1.27 (1.15,1.41)	1.03 (0.96,1.11)	0.89 (0.83,0.94)	1(Ref.)
Age ≥65	Non	1(Ref.)	0.91 (0.87,0.95)	1.05 (0.95,1.15)	1.07 (0.95,1.19)	1(Ref.)	0.90 (0.86,0.94)	1.03 (0.93,1.13)	1.05 (0.93,1.18)
	Light	0.97 (0.93,1.01)	0.79 (0.77,0.82)	0.84 (0.78,0.90)	0.94 (0.86,1.04)	1.20 (1.14,1.26)	1(Ref.)	1.07 (0.99,1.15)	1.21 (1.09,1.33)
	Moderate	1.15 (1.06,1.25)	0.85 (0.80,0.91)	0.84 (0.79,0.90)	0.98 (0.90,1.06)	1.34 (1.20,1.49)	1.01 (0.92,1.10)	1(Ref.)	1.15 (1.03,1.27)
	Heavy	1.36 (1.25,1.48)	1.05 (0.96,1.13)	0.91(0.85,0.99)	0.97 (0.92,1.02)	1.35 (1.22,1.49)	1.07 (0.98,1.18)	0.95 (0.86,1.04)	1(Ref.)
Men	Non	1(Ref.)	0.88 (0.85,0.91)	0.95 (0.89,1.02)	1.018(0.943,1.1)	1(Ref.)	0.88 (0.85,0.91)	0.95 (0.89,1.01)	1.03 (0.94,1.10)
	Light	0.93 (0.90,0.96)	0.75 (0.73,0.77)	0.78 (0.75,0.81)	0.89 (0.83,0.94)	1.23 (1.19,1.28)	1(Ref.)	1.04 (1.00,1.09)	1.18 (1.11,1.26)
	Moderate	1.12 (1.05,1.19)	0.82 (0.79,0.85)	0.80 (0.77,0.83)	0.90 (0.86,0.95)	1.40 (1.31,1.50)	1.02 (0.97,1.07)	1(Ref.)	1.13 (1.06,1.19)
	Heavy	1.29 (1.21,1.37)	0.99 (0.94,1.05)	0.85 (0.82,0.90)	0.95 (0.91,0.98)	1.34 (1.25,1.44)	1.04 (0.98,1.11)	0.90 (0.86,0.95)	1(Ref.)
Women	Non	1(Ref.)	0.96 (0.92,1.00)	1.12 (0.96,1.32)	1.02 (0.76,1.38)	1(Ref.)	0.96 (0.92,1.01)	1.13 (0.96,1.32)	1.03 (0.76,1.38)
	Light	1.03 (0.99,1.07)	0.94 (0.90,0.98)	1.10 (0.98,1.25)	1.28 (1.02,1.61)	1.12 (1.06,1.19)	1(Ref.)	1.17 (1.03,1.33)	1.37 (1.09,1.73)
	Moderate	1.21 (1.05,1.40)	1.11 (1.00,1.25)	1.03 (0.87,1.21)	1.25 (0.97,1.60)	1.23 (0.99,1.54)	1.08 (0.89,1.32)	1(Ref.)	1.22 (0.91,1.65)
	Heavy	0.96 (0.74,1.26)	1.09 (0.87,1.35)	1.00 (0.77,1.30)	1.11 (0.86,1.42)	0.82 (0.56,1.20)	0.94 (0.67,1.32)	0.87 (0.61,1.25)	1(Ref.)
Smokers	Non	1(Ref.)	0.93 (0.90,0.96)	1.05 (0.97,1.13)	1.12 (1.01,1.23)	1(Ref.)	0.92 (0.89,0.95)	1.02 (0.95,1.11)	1.08 (0.98,1.19)
	Light	1.00 (0.97,1.03)	0.82 (0.80,0.84)	0.88 (0.83,0.92)	0.97 (0.89,1.05)	1.20 (1.16,1.24)	1(Ref.)	1.08 (1.03,1.14)	1.20 (1.11,1.31)
	Moderate	1.17 (1.10,1.25)	0.91 (0.87,0.96)	0.86 (0.82,0.90)	0.94 (0.88,1.01)	1.35 (1.24,1.46)	1.06 (0.99,1.13)	1(Ref.)	1.11 (1.02,1.20)
	Heavy	1.34 (1.24,1.44)	1.07 (1.00,1.14)	0.91 (0.85,0.96)	0.99 (0.95,1.04)	1.31 (1.20,1.43)	1.06 (0.98,1.15)	0.91 (0.84,0.98)	1(Ref.)
Non-smoker	Non	1(Ref.)	0.89 (0.84,0.95)	0.89 (0.80,0.98)	0.93 (0.83,1.04)	1(Ref.)	0.89 (0.84,0.95)	0.89 (0.80,0.98)	0.93 (0.83,1.04)
	Light	0.87 (0.82,0.93)	0.74 (0.71,0.77)	0.73 (0.68,0.77)	0.85 (0.78,0.93)	1.16 (1.09,1.24)	1(Ref.)	0.99 (0.93,1.06)	1.16 (1.05,1.27)
	Moderate	1.11 (0.99,1.24)	0.76 (0.72,0.81)	0.77 (0.73,0.81)	0.89 (0.83,0.95)	1.44 (1.27,1.62)	0.99 (0.92,1.06)	1(Ref.)	1.15 (1.07,1.25)
	Heavy	1.19 (1.05,1.35)	0.93 (0.86,1.01)	0.82 (0.77,0.88)	0.91 (0.87,0.96)	1.32 (1.16,1.50)	1.03 (0.94,1.12)	0.90 (0.84,0.97)	1(Ref.)

aHR, adjusted hazard ratio; CI, confidence interval; IR, incidence rate.

Adjusted for age, sex, BMI, smoking status, physical activity, area of residence, income, hypertension, diabetes mellitus, dyslipidemia, systolic blood pressure, fasting glucose level, total cholesterol level, and serum creatinine level

REFERENCES

1. Abramson JL, Williams SA, Krumholz HM, et al. Moderate alcohol consumption and risk of heart failure among older persons. *Jama*. 2001;285:1971-7.

2. Aguilar D, Skali H, Moyé LA, et al. Alcohol consumption and prognosis in patients with left ventricular systolic dysfunction after a myocardial infarction. *J Am Coll Cardiol*. 2004;43:2015-21.

3. Bryson CL, Mukamal KJ, Mittleman MA, et al. The association of alcohol consumption and incident heart failure: the Cardiovascular Health Study. *J Am Coll Cardiol*. 2006;48:305-11.

4. Djoussé L, Gaziano JM. Alcohol consumption and risk of heart failure in the Physicians' Health Study I. *Circulation*. 2007;115:34-9.

5. Dorans KS, Mostofsky E, Levitan EB, et al. Alcohol and incident heart failure among middle-aged and elderly men: cohort of Swedish men. *Circ Heart Fail*. 2015;8:422-7.

6. Gemes K, Janszky I, Ahnve S, et al. Light-to-moderate drinking and incident heart failure--the Norwegian HUNT study. *Int J Cardiol*. 2016;203:553-60.

7. Goncalves A, Claggett B, Jhund PS, et al. Alcohol consumption and risk of heart failure: the Atherosclerosis Risk in Communities Study. *Eur Heart J*. 2015;36:939-45.

8. Janszky I, Ljung R, Ahnve S, et al. Alcohol and long-term prognosis after a first acute myocardial infarction: the SHEEP study. *Eur Heart J*. 2008;29:45-53.

9. Klatsky AL, Chartier D, Udaltsova N, et al. Alcohol drinking and risk of hospitalization for heart failure with and without associated coronary artery disease. *Am J Cardiol*. 2005;96:346-51.

10. Larsson SC, Orsini N, Wolk A. Alcohol consumption and risk of heart failure: a dose-response meta-analysis of prospective studies. *Eur J Heart Fail*. 2015;17:367-73.

11. Larsson SC, Wallin A, Wolk A. Contrasting association between alcohol consumption and risk of myocardial infarction and heart failure: Two prospective cohorts. *Int J Cardiol*. 2017;231:207-10.

12. Walsh CR, Larson MG, Evans JC, et al. Alcohol consumption and risk for congestive heart failure in the Framingham Heart Study. *Ann Intern Med*. 2002;136:181-91.

13. Wang Y, Tuomilehto J, Jousilahti P, et al. Lifestyle factors in relation to heart failure among Finnish men and women. *Circ Heart Fail*. 2011;4:607-12.

14. Wannamethee SG, Whincup PH, Lennon L, et al. Alcohol consumption and risk of incident heart failure in older men: a prospective cohort study. *Open Heart*. 2015;2.
