# Supplementary material for: Changes in Alcohol Consumption and Risk of Heart Failure: A Nationwide Population-Based Study in Korea
Source: Int J Environ Res Public Health. 2022 Dec 5;19(23):16265. doi: 10.3390/ijerph192316265 (PMC9736316; doi:10.3390/ijerph192316265)

Figure S1. Changes in alcohol consumption amount and risk of congestive heart failure by smoking status.

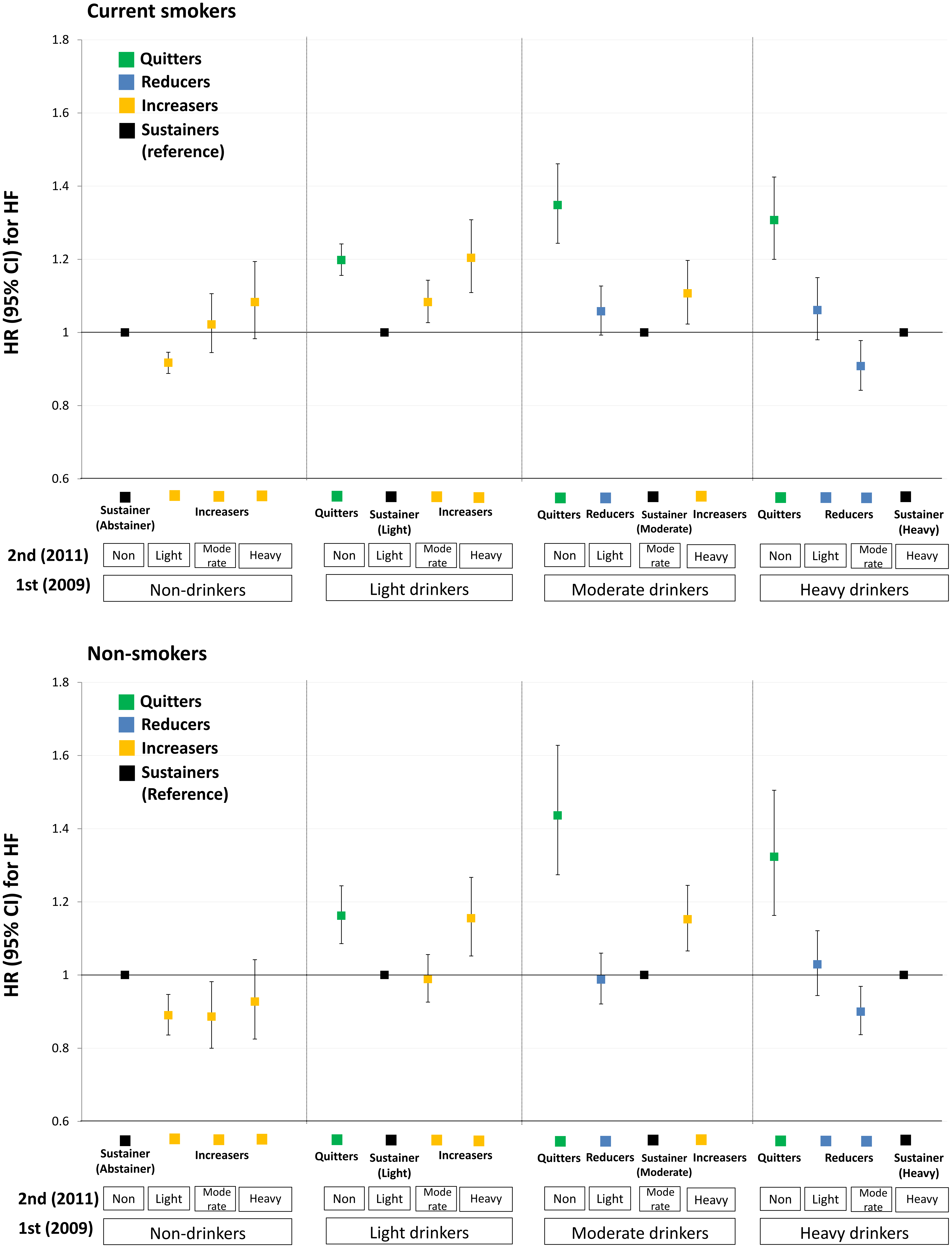

Supplement: Supplementary file 1 [file ijerph-19-16265-s001.zip › ijerph-1940159-supplementary.pdf]
